# Особенности гиперандрогении у мужчин

**DOI:** 10.14341/probl12732

**Published:** 2021-03-30

**Authors:** В. А. Филатова, Р. В. Роживанов

**Affiliations:** Национальный медицинский исследовательский центр эндокринологии; Национальный медицинский исследовательский центр эндокринологии

**Keywords:** гиперандрогения, тестостерон, дигидротестостерон, мужчины

## Abstract

ОБОСНОВАНИЕ. Сегодня проблема гиперандрогении изучается преимущественно по отношению к женщинам, у мужчин этот вопрос практически не затрагивается, в то же время гиперандрогения у них может быть ассоциирована с развитием ряда заболеваний.ЦЕЛЬ. Охарактеризовать варианты физиологической гиперандрогении у мужчин.МАТЕРИАЛЫ И МЕТОДЫ. Сплошное одномоментное исследование 100 мужчин с гиперандрогенией. При проведении исследования оценивались объем и структура простаты, объем яичек; определялись уровни лютеинизирующего гормона (ЛГ), общего тестостерона, глобулина, связывающего половые гормоны (ГСПГ), с дальнейшим расчетом уровня свободного тестостерона по Vermeullen, и дигидротестостерона (ДГТ). По результатам анализа гормонального статуса пациентов с гиперандрогенией были сформированы 4 группы пациентов: 1-я — пациенты с повышенным уровнем общего тестостерона и ГСПГ; 2-я — пациенты с повышенным уровнем общего тестостерона и нормальным уровнем ГСПГ; 3-я — пациенты с повышенным уровнем общего тестостерона, ДГТ при нормальном уровне ГСПГ; 4-я — пациенты с повышенным уровнем ДГТ при нормальном уровне общего тестостерона и ГСПГ. Статистически значимыми считали различия между группами при p<0,05.РЕЗУЛЬТАТЫ. Возраст и объем простаты пациентов 1-й группы были статистически значимо выше, чем в остальных группах. Для этой группы, несмотря на высокий уровень общего тестостерона, не было характерно наличие жалоб на акне. Пациенты 2-й группы чаще жаловались на акне, но распространенность этого симптома даже в этой группе являлась статистически значимо более низкой, чем у пациентов 3-й группы. При этом частота встречаемости алопеции была статистически значимо ниже во 2-й группе, чем у пациентов как 3-й, так и 4-й групп. Пациенты 3-й группы имели самые яркие клинические проявления гиперандрогении. Для 4-й группы была характерна алопеция.ЗАКЛЮЧЕНИЕ. Повышение уровня андрогенов может выявляться в любом возрасте. При этом у мужчин старшей возрастной группы повышение уровня общего тестостерона может быть обусловлено увеличением секреции ГСПГ и не сопровождаться повышением уровня свободного тестостерона. У молодых пациентов клинические проявления гиперандрогении могут отличаться: для пациентов с повышенным уровнем ДГТ характерна андрогенная алопеция; акне характерно для мужчин с повышенным уровнем общего и свободного тестостерона, а повышение ДГТ усугубляет эту проблему.

## Abstract (en)

BACKGROUND: Today the problem of hyperandrogenism in women is a widely studied and discussed while same issue in relation to men is barely raised. In clinical practice, hyperandrogenism can be the cause of a number of diseases.AIM: Provide characterization the variations of physiological hyperandrogenism in men.MATERIALS AND METHODS: Сontinuous cross-sectional study of 100 men with hyperandrogenism. The study assessed the volume and structure of the prostate, the volume of the testicles; the levels of luteinizing hormone (LH), total testosterone, sex hormone binding globulin (SHBG) were determined with further calculation of the level of free testosterone according to Vermeullen, and dihydrotestosterone (DHT). Based on the results of the analysis of the hormonal status of patients with hyperandrogenism, 4 groups of patients were formed: 1-patients with increased total testosterone and SHBG levels; 2-patients with elevated total testosterone levels and normal SHBG levels; 3-patients with an increased level of total testosterone, DHT with a normal level of SHBG; 4-patients with an increased level of DHT with normal levels of total testosterone and SHBG. The difference between groups of patients was determined, a p-value <0.05 was considered statistically significant.RESULTS: The age and volume of the prostate in group 1 patients were statistically significantly higher than in the other groups. This group, despite the high level of total testosterone, was not characterized by complaints of acne. Group 2 patients complained of acne more often, but the prevalence of this symptom even in this group was statistically significantly lower than in group 3 patients. At the same time, the frequency of occurrence of alopecia was statistically significantly lower in group 2 than in patients of both groups 3 and 4. Patients of group 3 had the most striking clinical manifestations of hyperandrogenism. Group 4 was characterized by alopecia.CONCLUSION: An increase of androgen levels can be detected at any age. At the same time, in men of the older age group, an increase in the level of total testosterone may be due to an increase in the secretion of SHBG and not be accompanied by an increase in the level of free testosterone. In young patients, the clinical manifestations of hyperandrogenism may differ: patients with elevated DHT levels are characterized by androgenic alopecia; acne is common in men with elevated total and free testosterone levels, and increased DHT exacerbates the problem.

## ОБОСНОВАНИЕ

Термин «гиперандрогения» подразумевает избыточный синтез андрогенных гормонов [1]. Несмотря на то что подобное состояние может развиваться у лиц обоего пола, в настоящее время это понятие используется преимущественно по отношению к женщинам. Сегодня проблема гиперандрогении у женщин — широко изучаемый и обсуждаемый вопрос, затрагивающийся на каждой конференции репродуктивного здоровья, в то время как по отношению к мужчинам этот вопрос практически не затрагивается.

Если рассматривать гиперандрогению как самостоятельную нозологическую единицу, то можно условно выделить физиологическую гиперандрогению — обусловленную гиперпродукцией тестостерона и/или дигидротестостерона (ДГТ) при нормальном уровне лютеинизирующего гормона (ЛГ) и патологическую — сопровождающуюся подавлением ЛГ, обусловленным серьезной сопутствующей соматической патологией надпочечников, яичек или приемом лекарственных средств с андрогенным эффектом, анаболических стероидов [2]. Отдельным видом гиперандрогении является повышение андрогенов у мужчин с андрогенпродуцирующими опухолями яичек или надпочечников [3][4]. В нашем исследовании охарактеризованы варианты физиологической гиперандрогении у мужчин.

## ЦЕЛЬ ИССЛЕДОВАНИЯ

Охарактеризовать варианты физиологической гиперандрогении у мужчин.

## МАТЕРИАЛЫ И МЕТОДЫ

Место и время проведения исследования

ФГБУ «НМИЦ эндокринологии» Министерства здравоохранения РФ, Москва. Исследование выполнено в период с сентября 2020 г. по январь 2021 г.

Изучаемые популяции

Формирование групп проводилось из пациентов, обратившихся за медицинской помощью в ФГБУ «НМИЦ эндокринологии» Министерства здравоохранения РФ.

Критериями включения являлись мужской пол; возраст старше 18 лет; повышенные уровни общего тестостерона и/или ДГТ; нормальный уровень ЛГ.

Критериями невключения являлись новообразования гипоталамо-гипофизарной области, яичек, надпочечников; врожденная дисфункция коры надпочечников; использование любых препаратов из групп антиэстрогенов, эстрогенов, гестагенов, ингибиторов ароматазы, антиандрогенов, гонадотропинов, анаболических стероидов, ингибиторов стероидогенеза; алкоголизм/наркомания; сахарный диабет 1 и 2 типов; синдром гиперкортицизма.

Критерии исключения — не предусматривались.

Всего в исследование были включены 100 пациентов, возраст 26 (минимум 18; максимум 58) лет.

Способ формирования выборки из изучаемой популяции

Выборка формировалась сплошным способом.

Дизайн исследования

Сплошное одномоментное исследование мужчин с повышенным уровнем общего тестостерона и/или ДГТ.

Методы

При проведении исследования оценивались объем и структура простаты, объем яичек с помощью УЗИ на аппарате Aplio 500 № 416508; определялись уровни ЛГ (норма 2,5–11,0 ЕД/л), общего тестостерона (норма 12,0–30,0 нмоль/л), глобулина, связывающего половые гормоны (ГСПГ) (норма 12–65 нмоль/л), ДГТ (норма 250–990 пг/мл) на автоматическом анализаторе Vitros ECi (Johnson and Johnson (Великобритания)) методом усиленной хемилюминесценции. Уровень свободного тестостерона определялся расчетным методом по Vermeullen (норма 243–900 пмоль/л) [5]. Анализ гормонального статуса пациентов с гиперандрогенией позволил сформировать 4 группы пациентов по лабораторным признакам.

1. Пациенты с повышенным уровнем общего тестостерона и ГСПГ (n=12).

2. Пациенты с повышенным уровнем общего тестостерона и нормальным уровнем ГСПГ (n=15).

3. Пациенты с повышенным уровнем общего тестостерона, ДГТ при нормальном уровне ГСПГ (n=11).

4. Пациенты с повышенным уровнем ДГТ при нормальных уровнях общего тестостерона и ГСПГ (n=62).

Статистический анализ

Принципы расчета размера выборки

Исследование пилотное. Размер выборки предварительно не рассчитывался.

Методы статистического анализа данных

Полученные данные обработаны с использованием пакета статистических программ STATISTICA 13.0 (StatSoft Inc., США) [6]. Различия между группами определялись с использованием U-критерия Манна–Уитни для количественных признаков и точного критерия Фишера — для качественных. Различия между группами считались статистически значимыми при р<0,05.

Этическая экспертиза

Исследование одобрено Локальным этическим комитетом ФГБУ «НМИЦ эндокринологии» Минздрава России (протокол № 17 от 28.10.2020).

## РЕЗУЛЬТАТЫ

При сравнении групп были выявлены статистически значимые различия в ряде показателей (табл. 1). Первая группа пациентов статистически значимо отличалась от остальных групп пациентов более старшим возрастом, также для этих пациентов было характерно наличие избыточной массы тела. Объем простаты мужчин этой группы статистически значимо превышал таковой у мужчин других групп. Кроме того, для этой группы пациентов, несмотря на высокий уровень общего тестостерона (уровень свободного тестостерона был статистически значимо ниже по сравнению с таковым у пациентов 2-й и 3-й группы, но выше, чем у мужчин 4-й группы), не было характерно наличие жалоб на акне. Алопеция также встречалась редко.

**Table:** Таблица 1. Характеристика групп пациентов с гиперандрогенией. Представлены медианы и границы интерквартильного отрезка

Параметр	1 гр. (n=12)	2 гр. (n=15)	3 гр. (n=11)	4 гр. (n=62)	1–2	1–3	1–4	2–3	2–4	3–4
Возраст	50[ 46; 55 ]	23[ 20; 25 ]	23[ 21; 25 ]	27[ 25; 28 ]	<0,001	<0,001	<0,001	0,83	<0,001	<0,001
ИМТ, кг/м2	27,2[ 26,3; 27,8 ]	24,2[ 23,2; 26,4 ]	24,1[ 22,9; 25,4 ]	24,8[ 23,2; 27,1 ]	0,002	0,006	0,005	0,85	0,47	0,54
Билатеральный объем яичек, мл	20[ 18; 22 ]	22[ 19; 24 ]	21[ 20; 23 ]	22[ 20; 23 ]	0,34	0,41	0,22	0,79	0,92	0,77
Объем простаты, мл	26[ 24; 29 ]	18[ 16; 19 ]	20[ 18; 22 ]	18[ 17; 21 ]	<0,001	<0,001	<0,001	0,07	0,60	0,12
ЛГ, ЕД/л	3,7[ 3,0; 4,5 ]	4,2[ 3,3; 5,0 ]	4,0[ 2,9; 4,6 ]	3,9[ 3,1; 4,9 ]	0,51	0,83	0,61	0,44	0,69	0,49
ГСПГ, нмоль/л	79,4[ 74,7; 91,1 ]	32,9[ 26,7; 45,3 ]	30,6[ 23,2; 38,1 ]	29,1[ 23,4; 37,8 ]	<0,001	<0,001	<0,001	0,39	0,18	0,88
Общий тестостерон, нмоль/л	38,2[ 36,1; 42,9 ]	36,2[ 34,9; 38,2]	38,1[ 34,9; 39,4 ]	15,8[ 13,6; 19,3 ]	0,09	0,41	<0,001	0,21	<0,001	<0,001
Свободный тестостерон, пмоль/л	505[ 421; 590 ]	929[ 757; 1000 ]	1000[ 910; 1100 ]	369[ 288; 448 ]	<0,001	<0,001	<0,001	0,148	<0,001	<0,001
ДГТ, пг/мл	700[ 645; 836 ]	789[ 720; 890 ]	2805[ 1980; 3621 ]	2496[ 2208; 2896 ]	0,06	<0,001	<0,001	0,001	<0,001	0,26
Акне, %	0	67	100	31	<0,001	<0,001	0,029	0,05	0,016	<0,001
Алопеция %	25	13	82	93	0,63	0,012	<0,001	<0,001	<0,001	0,22
Изменения простаты объемные, %	8	0	0	0	<0,001	0,001	<0,001	1,0	1,0	1,0
Изменения простаты диффузные, %	25	13	18	18	0,63	1,0	0,69	1,0	1,0	1,0

Напротив, пациенты 2-й группы предъявляли жалобы на акне, но распространенность этого симптома даже в этой группе являлась статистически значимо более низкой, нежели у пациентов 3-й группы. При этом частота встречаемости алопеции была существенно ниже, чем у пациентов как 3-й, так и 4-й групп.

Пациенты с повышенным уровнем общего тестостерона и ДГТ (3-я группа) имели самые яркие клинические проявления гиперандрогении. Все пациенты из этой группы предъявляли жалобы на наличие акне, а также на усиленное выпадение волос на голове.

Для пациентов 4-й группы также была характерна алопеция. Жалобы на наличие акне присутствовали, но их частота статистически значимо различалась по сравнению с 1-й группой в сторону увеличения, а по сравнению с другими группами — в сторону уменьшения. Для мужчин 4-й группы были характерны статистически значимо меньшие уровни свободного тестостерона по сравнению с пациентами других групп.

## ОБСУЖДЕНИЕ

Репрезентативность выборок

Оценить репрезентативность выборки по отношению к общей популяции не представляется возможным, поскольку формирование выборки проводилось сплошным методом из пациентов, наблюдавшихся только в федеральном научном центре.

Сопоставление с другими публикациями

В клинической практике гиперандрогения может быть причиной развития ряда заболеваний [7]. Повышение уровня тестостерона, ДГТ или генетически обусловленная чувствительность рецепторов к андрогенам может приводить к развитию такого заболевания, как андрогенная алопеция [8]. В нашем исследовании она была выявлена у мужчин с повышенным уровнем ДГТ вне зависимости от уровня тестостерона, что согласуется с современной концепцией влияния повышенного уровня ДГТ на развитие андрогенной алопеции [9]. Так, в исследовании H. Schweikert и соавт. было продемонстрировано, что волосяные фолликулы и кожа волосистой части головы больных андрогенной алопецией содержат ДГТ в более высоких концентрациях, чем образцы здоровых тканей [10]. При определении концентрации ДГТ и тестостерона у 52 пациентов обоих полов с ранней андрогенной алопецией статистически значимого повышения обнаружено не было, однако авторы выявили существенное увеличение соотношения ДГТ/тестостерон [11].

Еще одним заболеванием, в развитии которого большое значение может иметь гиперандрогения, является угревая болезнь [12]. Под воздействием андрогенов увеличивается объем кожного сала, в котором снижается концентрация незаменимой альфа-линоленовой кислоты — основного регулятора дифференцировки кератиноцитов протока сально-волосяного фолликула, что проявляется появлением открытых и закрытых комедонов и создает благоприятные условия для размножения микрофлоры [13]. По результатам проведенного исследования акне было характерно для мужчин с повышенным уровнем общего и свободного тестостерона, при этом наличие повышенного уровня ДГТ приводило к увеличению распространенности акне, вплоть до 100% случаев. Однако эта ситуация не распространялась на мужчин из 1-й группы, поскольку у них повышенный уровень общего тестостерона, строго говоря, не был признаком гиперандрогении, а обусловлен повышением уровня ГСПГ. При этом уровень свободного тестостерона, который отражает степень андрогенной насыщенности организма, был статистически значимо ниже, чем наблюдаемый во 2-й и 3-й группах. Хотя уровень этого показателя статистически значимо превышал таковой в 4-й группе, величины уровня свободного тестостерона, полученные у мужчин как 1-й, так и 4-й групп, находились в пределах референсных значений. Повышение уровня ГСПГ характерно для мужчин старшей возрастной группы, при этом в пожилом возрасте у мужчин оно чаще приводит к развитию дефицита тестостерона [14]. В случае если у пациентов с возрастом секреция общего тестостерона не нарушается, то и повышение ГСПГ не приводит к дефициту свободного тестостерона — он остается в пределах референсных значений.

Характерным признаком пациентов 1-й группы также было увеличение объема предстательной железы, что может объясняться возрастом пациентов. Исследователями было продемонстрировано наличие ассоциации между возрастом мужчины и развитием доброкачественной гиперплазии предстательной железы [15]. У молодых пациентов, включенных в наше исследование, несмотря на повышенные уровни андрогенов, каких-либо изменений объема или структуры предстательной железы выявлено не было.

Клиническая значимость результатов

Полученные результаты имеют значение для клинической практики, поскольку позволяют производить дифференциальную диагностику различных вариантов гиперандрогении, что необходимо для персонификации лечения. Выявление при обследовании пожилых мужчин повышенного уровня общего тестостерона обуславливает необходимость оценки уровня ГСПГ с дальнейшим расчетом уровня свободного тестостерона, что при получении величин этого показателя, укладывающихся в референсный интервал, позволит исключить гиперандрогению у пациента.

Ограничения исследования

Ограничениями исследования являются проблемы с репрезентативностью выборки в отношении общей популяции (сформирована только из пациентов крупного федерального центра), а также использование в исследовании не прямого метода определения свободного тестостерона, а расчетной методики.

Направления дальнейших исследований

В продолжение проведенного исследования планируется изучить факторы риска, характерные для разных вариантов гиперандрогений, связанные со стероидогенезом.

## ЗАКЛЮЧЕНИЕ

Повышение уровня андрогенов может выявляться в любом возрасте. При этом у мужчин старшей возрастной группы повышение уровня общего тестостерона может не свидетельствовать о гиперандрогении, а обусловливаться увеличением секреции ГСПГ и не сопровождаться повышением уровня свободного тестостерона. У молодых пациентов клинические проявления гиперандрогении зависят от ее варианта. Так, для пациентов с повышенным уровнем ДГТ была характерна андрогенная алопеция. Акне было характерно для мужчин с повышенным уровнем общего и свободного тестостерона, хотя повышение ДГТ также усугубляло эту проблему.
